# Study on the clinical characteristics, treatment, and outcome influencing factors of severe pneumonia complicated with ARDS

**DOI:** 10.1097/MD.0000000000040316

**Published:** 2024-11-08

**Authors:** Wei Zhang, Han Xiao, Xiaowei Tong, Lan He, Xinjuan Xu, Jiulong Dong

**Affiliations:** a Department of Critical Care Medicine, The Third People’s Hospital of Yichang Hubei, Yichang, Hubei, China; b Department of Pulmonary and Critical Care Medicine, The Third People’s Hospital of Yichang Hubei, Yichang, Hubei, China; c Department of Internal medicine, The Third People’s Hospital of Yichang Hubei, Yichang, Hubei, China.

**Keywords:** acute respiratory distress syndrome, influencing factors, prognosis, severe pneumonia

## Abstract

To investigate the clinical characteristics, treatment methods, and factors influencing the prognosis of patients with severe pneumonia complicated by Acute Respiratory Distress Syndrome (ARDS), aiming to provide references for clinical decision-making and improve patient outcomes. A retrospective analysis was conducted on 118 patients with severe pneumonia complicated by ARDS treated at our hospital from June 2018 to December 2022. Based on treatment outcomes, patients were divided into a death group (n = 75) and a survival group (n = 43). General data and clinical laboratory indicators, including blood urea nitrogen, serum creatinine, C-reactive protein, procalcitonin, arterial partial pressure of oxygen, and arterial partial pressure of carbon dioxide, were collected and compared between the 2 groups to identify independent factors affecting prognosis. Among the 118 patients, the mortality rate was 63.56%. Patients in the death group had a significantly higher average age (57.15 ± 13.38 years) and a higher proportion of severe ARDS (66.67%) compared to the survival group (40.02 ± 11.41 years, 30.23%, *P* < .001). The death group had significantly lower white blood cell counts (8.10 ± 1.64 × 10^9^/L), oxygenation index (19.82 ± 2.29), and duration of mechanical ventilation (7.79 ± 2.11 days) compared to the Survival group (8.92 ± 1.22 × 10^9^/L, 13.42 ± 1.82, 12.23 ± 3.05 days, *P* < .05). Conversely, the death group had significantly higher levels of blood urea nitrogen (6.87 ± 1.80 mmol/L), C-reactive protein (130.55 ± 50.28 mg/L), procalcitonin (5.50 ± 2.11 ng/mL), arterial partial pressure of carbon dioxide (41.12 ± 5.56 mm Hg), and a higher proportion of viral infections (48.00%) compared to the survival group (5.90 ± 1.72 mmol/L, 101.77 ± 55.56 mg/L, 3.98 ± 1.15 ng/mL, 35.59 ± 6.22 mm Hg, 27.91%, *P* < .05). Logistic regression analysis revealed that age (odds ratios [OR] = 1.990, 95% confidence interval [CI]: 1.306–3.033, *P* < .001), oxygenation index (OR = 1.426, 95% CI: 1.123–1.649, *P* < .001), and duration of mechanical ventilation (OR = 0.694, 95% CI: 0.557–0.864, *P* < .001) were independent factors influencing patient prognosis. This indicates that an increase in age and a decrease in oxygenation index are associated with a significantly higher risk of mortality, while shorter mechanical ventilation duration is related to poorer prognosis. Advanced age, lower oxygenation index, and shorter duration of mechanical ventilation are unfavorable prognostic factors in patients with severe pneumonia complicated by ARDS. These findings aid clinicians in identifying high-risk patients, optimizing treatment plans, and improving patient prognosis.

## 1. Introduction

Severe pneumonia is an advanced stage of pneumonia. As the condition progresses, patients may develop Acute Respiratory Distress Syndrome (ARDS), leading to respiratory failure, shock, sepsis, multiple organ failure, and even death.^[1,2]^ The condition of severe pneumonia combined with ARDS is complex, recovery time is prolonged, and the prognosis is poor. Identifying risk factors affecting the prognosis of patients with severe pneumonia combined with ARDS and providing certain interventions can help improve patient outcomes.^[3,4]^

There are many studies on the prognostic factors of severe pneumonia complicated by ARDS. Some studies have found that the mortality rate of patients with viral infections is higher than those with fungal and bacterial infections.^[5,6]^ Other studies suggest that age, hospital stay, and duration of mechanical ventilation are important factors affecting patient prognosis.^[7,8]^ However, the results of these studies are not entirely consistent, which limits their practical clinical guidance. Therefore, this study aims to explore the prognostic factors of severe pneumonia complicated by ARDS in hopes of providing references for clinical practice. The report is as follows.

## 2. Materials and methods

### 2.1. General data

This study was approved by The Ethics Committee of The Third People’s Hospital of Yichang Hubei (No. 2024-0406-k45). Hundred eighteen cases of patients with severe pneumonia complicated by ARDS were selected by using a retrospective study method, who were treated at our hospital from June 2018 to December 2022. This retrospective cohort study was designed to analyze the clinical features, treatment methods, and prognostic factors of 118 patients with severe pneumonia complicated with ARDS who were treated in our hospital from June 2018 to December 2022. Patients were divided into 2 groups according to different outcomes. Death group and survival group. Inclusion criteria: (1) The diagnosis of severe pneumonia meets the criteria set by the American Thoracic Society/American Infectious Diseases Society,^[9]^ a diagnosis of severe pneumonia can be made if a patient meets at least 1 primary criterion or 3 or more secondary criteria. Primary criteria: 1. Endotracheal intubation requiring mechanical ventilation. 2. Septic shock requiring vasoactive agents despite aggressive fluid resuscitation. Secondary criteria: 1. Respiratory rate ≥ 30 breaths per minute. 2. Partial pressure of oxygen (PaO_2_)/FiO_2_ ≤ 250 mm Hg. 3. Multilobar infiltration. 4. Altered mental status and/or disorientation. 5. Blood urea nitrogen (BUN) ≥ 20 mg/dL. 6. Leukopenia (white blood cells [WBC] < 4 × 10^9^/L). 7. Thrombocytopenia (platelets < 100 × 10^9^/L). 8. Hypothermia (core body temperature < 36 °C). 9. Hypotension requiring fluid resuscitation. And ARDS conforms to the ARDS Berlin diagnostic criteria established by the European Society of Intensive Care Medicine.^[10]^ ARDS is an acute, diffuse, and inflammatory lung injury induced by risk factors such as pneumonia, non-pulmonary infection, trauma, blood transfusion, burn, aspiration, or shock. (2) Age over 18 years. (3) Complete preservation of clinical data. Exclusion criteria: (1) Presence of other significant organ diseases such as liver or kidney diseases. (2) Presence of cardiovascular or cerebrovascular diseases. (3) Presence of malignant tumors or other severe illnesses. (4) Patients with other types of lung disease before the onset of severe pneumonia. (5) Patients with malignant tumor. (6) Patients receiving immunosuppressive therapy.

### 2.2. Data collecting

The patient information was collected retrospectively through the case system. The patients’ gender, age, body mass index, presence of hypertension, duration of diabetes, duration of mechanical ventilation, and the grading of acute respiratory distress syndrome were recorded. All patients underwent bronchoscopy, and secretions were collected for culture. The specimens were cultured and pathologically examined using the BS100 fully automatic bacterial culture detection system from Zhuhai Beso Biotechnology Co., Ltd. In the morning, fasting peripheral venous blood was drawn from the patients, and routine blood counts including WBC, platelets, albumin, and hemoglobin were measured using the MACCURA F810 fully automatic blood cell analyzer. Kidney function indicators such as BUN, serum creatinine, and C-reactive protein (CRP) were tested using the LABOSPECT 008 α fully automatic biochemical analyzer from Hitachi and its matching reagents. Procalcitonin (PCT) was measured using the Q20 fully automatic fluorescence immunoassay analyzer from Zhongyuan Huiji Co. Radial artery blood was drawn from the patients to measure the PaO_2_ and arterial partial pressure of carbon dioxide (PaCO_2_), and the oxygenation index was calculated as (mean airway pressure × inspired oxygen concentration/ arterial oxygen partial pressure) × 100. All experimental data are carried out through uniform equipment and standard operating procedures to ensure data accuracy and comparability. Laboratory tests and blood gas analysis are performed while the patient is fasting, ensuring that the data is not affected by recent diet.

### 2.3. Statistical analysis

Data analysis was performed using SPSS22.0 software. Age, blood routine indicators and other data were expressed as (‾χ± s), and t-test was used for comparison between groups. Data such as gender were expressed as frequency or percentage, and χ^2^ test was used for comparison between groups. Logistic regression analysis was used to analyze the influencing factors of prognosis. *P* < .05 indicated that the difference was statistically significant.

## 3. Results

### 3.1. Prognosis and etiology

Among 118 patients, 75 patients died after treatment, with a mortality rate of 63.56%. Etiological examination revealed: 48 cases of viral infections, 42 cases of bacterial infections, 20 cases of fungal infections, and 8 cases of mixed infections.

### 3.2. Comparison of clinical general data between dead and surviving patients

Patients in the deceased group were older than those in the survival group (*P* < .05); the proportion of patients with severe ARDS in the deceased group was significantly higher than that in the survival group (*P* < .05). See Table 1.

**Table 1:** Comparison of clinical general data between dead and surviving patients.

Clinical data	Death group (n = 75)	Survival group (n = 43)	t/□^□^	*P*
Gender				
Male	42 (56.00)	24 (55.81)	0.000	.984
Female	33 (44.00)	19 (44.19)		
Age (years)	57.15 ± 13.38	40.02 ± 11.41	7.050	<.001
Body mass index (kg/m^2^)	22.12 ± 3.03	22.09 ± 2.87	0.053	.958
Diabetes (%)	11 (14.367)	6 (13.95)	0.011	.915
Hypertension (%)	22 (29.33)	14 (32.56)	0.134	.714
ARDS classification				
Mild to moderate	25 (33.33)	30 (69.77)	14.579	<.001
Severe	50 (66.67)	13 (30.23)		

### 3.3. Comparison of laboratory indexes between dead and surviving patients

In the deceased group, patients had significantly lower WBC counts, oxygenation indices, and mechanical ventilation times compared to the survival group (*P* < .05). Conversely, BUN, CRP, PCT, PaCO_2_, and the proportion of viral infections were significantly higher than in the survival group (*P* < .05). See Table 2.

**Table 2:** Comparison of laboratory indexes between dead and surviving patients.

Clinical data	Death group (n = 75)	Survival group (n = 43)	t/□^□^	*P*
WBC (×10^9^/L)	8.10 ± 1.64	8.92 ± 1.22	‐2.855	.005
PLT (×10^9^/L)	165.56 ± 53.32	178.81 ± 60.03	‐1.240	.217
Alb (g/L)	30.03 ± 4.69	28.89 ± 5.10	1.231	.221
Hb (g/L)	120.03 ± 20.15	117.65 ± 18.89	0.631	.529
BUN (mmol/L)	6.87 ± 1.80	5.90 ± 1.72	2.863	.005
Scr (□mol/L)	121.03 ± 45.55	108.46 ± 41.11	1.494	.138
CRP (mg/L)	130.55 ± 50.28	101.77 ± 55.56	2.879	.005
PCT (ng/mL)	5.50 ± 2.11	3.98 ± 1.15	4.362	<.001
PaCO_2_ (mm Hg)	41.12 ± 5.56	35.59 ± 6.22	4.978	<.001
PaO_2_ (mm Hg)	58.82 ± 7.98	60.03 ± 8.11	‐0.788	.432
Oxygenation index	19.82 ± 2.29	13.42 ± 1.82	15.695	<.001
Mechanical ventilation time (d)	7.79 ± 2.11	12.23 ± 3.05	‐9.136	<.001
Virus infection (%)	36 (48.00)	12 (27.91)	4.573	.032

Alb = albumin, BUN = blood urea nitrogen, CRP = C-reactive protein, Hb = haemoglobin, PaCO2 = arterial partial pressure of carbon dioxide, PaO2 = arterial partial pressure of oxygen, PLT = platelets, PCT = procalcitonin, Scr = serum creatinine, WBC = white blood cells.

### 3.4. Multiple-factor analysis

Using statistically significant indicators as independent variables and prognosis (death = 1, survival = 0) as the dependent variable, a logistic regression analysis was conducted. The results indicated that age, oxygenation index, and mechanical ventilation time are significant factors affecting patient outcomes, with odds ratios (OR) of 1.990, 1.426, and 0.361, respectively, and all were significant (*P* < .05). See Table 3.

**Table 3:** Multiple-factor analysis.

Factor	□β	SE	Walds	*P*	OR (95% CI)
Age	0.688	0.215	10.240	<.001	1.990 (1.306–3.033)
Oxygenation index	0.308	0.098	9.878	<.001	1.361 (1.123–1.649)
Mechanical ventilation time	‐0.366	0.112	10.679	<.001	0.694 (0.557–0.864)

## 4. Discussion

ARDS is a severe form of acute respiratory failure caused by a variety of diseases, with severe pneumonia being the most common primary condition. Severe pneumonia combined with ARDS constitutes a group of severe clinical syndromes that rapidly progress and are associated with a high risk of death.^[11,12]^ Studies have found that the mortality rate for patients with severe pneumonia combined with ARDS can be as high as 68.13%.^[13,14]^ This study found that among 118 patients, 75 died after treatment, resulting in a mortality rate of 63.56%. This outcome is largely consistent with previous clinical research.^[15,16]^

Improving the success rate of treatment for severe pneumonia combined with ARDS is a current hotspot in clinical research. There are many studies on risk factors affecting patient prognosis, though their results are not always consistent.^[17,18]^ Research has identified that a decline in PaO_2_/FiO_2_ and older age are risk factors that increase mortality.^[19,20]^ Deceased patients tend to be older, have a higher proportion of severe ARDS, lower oxygenation indices, shorter mechanical ventilation times, and higher PaCO_2_ levels. This pattern may be attributed to the relatively poorer immune system function in older patients, which leads to decreased defense against pathogenic microbes and a lower tolerance to hypoxic conditions.^[21,22]^ Severe ARDS is particularly critical and often accompanied by disturbances in consciousness, which increases the difficulty of treatment.^[23,24]^ Patients with low oxygenation indices, shorter mechanical ventilation times, and higher PaCO_2_ levels typically do not achieve optimal mechanical ventilation effects and are unable to effectively correct the hypoxic state of the body. Persistent hypoxia can lead to dysfunction of vital organs, ultimately resulting in the patient’s death.^[25,26]^

White blood cells are crucial immune cells that defend against pathogens and secrete cytokines like interleukins, interferons, and tumor necrosis factors, which participate in the immune response.^[27,28]^ BUN is a sensitive indicator of renal function, and impaired renal function in patients with severe pneumonia combined with ARDS suggests the potential for multi-organ damage.^[29,30]^ CRP and PCT are sensitive markers of inflammation, with PCT also being useful for differentiating between viral and bacterial infections.^[31,32]^ This study found that, compared to surviving patients, deceased patients had lower WBC counts and higher levels of BUN, CRP, PCT, and the proportion of viral infections. The decrease in WBCs reflects a weakened immune response, reducing the body’s defense against pathogens. Elevated levels of BUN, CRP, and PCT indicate a more severe infection and inflammatory response, possibly pointing to multi-organ dysfunction syndrome, which is associated with a poorer prognosis. Currently, clinical diagnosis often faces challenges with low detection rates for many viruses, making it difficult to obtain accurate etiological evidence. Typically, diagnosis relies on patient history, clinical symptoms, physical signs, and trial medication. However, treatment outcomes are often not ideal. Moreover, viral infections can damage airway epithelium and expose and upregulate receptors, increasing the risk of secondary infections and complicating the clinical picture and treatment.^[33–35]^

The results of the logistic regression analysis in this study indicated that age, oxygenation index, and duration of mechanical ventilation are factors influencing patient prognosis. This suggests that in patients with severe pneumonia complicated by ARDS, older age, lower oxygenation index, and shorter mechanical ventilation duration are risk factors for poor prognosis. In future clinical practice, these patients should be considered high-risk.

Patients with severe pneumonia complicated by ARDS face critical conditions and high mortality rates, making it crucial to identify risk factors affecting their prognosis. Although there are numerous clinical studies on the prognostic factors for patients with severe pneumonia and ARDS, the results are not always consistent. It has been found that older age, lower oxygenation index, and shorter mechanical ventilation duration are risk factors for poor prognosis. Clinically, it is important to recognize these high-risk factors and mitigate risks to enhance treatment outcomes for patients. Older patients have weaker immune function and reduced tolerance, leading to an increased risk of worsening the condition. A lower oxygenation index reflects severely impaired lung function, making it more difficult for patients to regain normal respiratory function, while shorter periods of mechanical ventilation may mean that patients are removed from respiratory support when their vital signs are unstable, increasing the risk of death. Multivariate regression analysis showed that the risk of death increased about twice for each additional year of age (OR = 1.990), suggesting a need for more aggressive interventions in older patients. For each unit increase in the oxygenation index, the risk of death increased by about 1.426 times, suggesting that the improvement of oxygenation status is essential to improve patient survival. Shortening of mechanical ventilation duration was associated with a reduced risk of death (OR = 0.694), suggesting that timely and effective management of mechanical ventilation has a positive impact on patient prognosis. In comparison with existing studies, we found that factors such as patient age, oxygenation index, and duration of mechanical ventilation play significant roles in the prognosis of patients with severe pneumonia complicated by ARDS, which aligns with findings reported in other research. However, our study also discovered that an increased proportion of viral infections is significantly associated with poor patient outcomes, a finding not widely reported in prior literature. Additionally, we observed that the white blood cell count was significantly lower in the deceased group, which may reflect a different immune response pattern. This observation has yielded mixed results in related studies. These differences suggest that our understanding of the disease mechanisms requires further exploration, offering new directions for future research.

This study has several limitations that should be acknowledged. First, the retrospective design inherently carries the risk of selection bias and information bias, as the data were collected from medical records that may not have captured all relevant variables comprehensively. Second, the study was conducted in a single tertiary hospital, which may limit the generalizability of the findings to other settings or populations with different demographic and clinical characteristics. Third, the sample size of 118 patients, while sufficient to identify significant associations, may not be large enough to detect smaller effect sizes or to perform more detailed subgroup analyses. Additionally, the study did not account for all potential confounding factors, such as variations in treatment protocols, underlying comorbidities beyond those included in the exclusion criteria, and the timing of interventions, which could influence patient outcomes. Future studies should consider a multicenter, large sample size prospective design to validate the findings of this study. In addition, further exploration of other potential prognostic factors, such as markers of immune function and therapeutic interventions. It will help to understand additional prognostic factors in severe pneumonia complicated with ARDS and explore additional prognostic factors in severe pneumonia complicated with ARDS.

In summary, patients with severe pneumonia complicated by ARDS have a high mortality rate, and their treatment outcomes are influenced by factors such as age, oxygenation index, and duration of mechanical ventilation.

## Author contributions

**Conceptualization:** Wei Zhang, Han Xiao, Xiaowei Tong, Lan He, Xinjuan Xu, Jiulong Dong.

**Data curation:** Wei Zhang, Han Xiao, Xiaowei Tong, Lan He, Xinjuan Xu, Jiulong Dong.

**Formal analysis:** Wei Zhang, Han Xiao, Xiaowei Tong, Lan He, Xinjuan Xu.

**Funding acquisition:** Jiulong Dong.

**Investigation:** Wei Zhang, Han Xiao, Xiaowei Tong, Lan He, Xinjuan Xu, Jiulong Dong.

**Methodology:** Wei Zhang, Han Xiao, Xiaowei Tong, Lan He, Xinjuan Xu, Jiulong Dong.

**Supervision:** Lan He.

**Validation:** Xiaowei Tong, Lan He.

**Writing – original draft:** Wei Zhang, Jiulong Dong.

**Writing – review & editing:** Wei Zhang, Jiulong Dong.
